# Artificial Intelligence in the Management of Glioma: Era of Personalized Medicine

**DOI:** 10.3389/fonc.2019.00768

**Published:** 2019-08-14

**Authors:** Houman Sotoudeh, Omid Shafaat, Joshua D. Bernstock, Michael David Brooks, Galal A. Elsayed, Jason A. Chen, Paul Szerip, Gustavo Chagoya, Florian Gessler, Ehsan Sotoudeh, Amir Shafaat, Gregory K. Friedman

**Affiliations:** ^1^Department of Neuroradiology, University of Alabama, Birmingham, AL, United States; ^2^Russell H. Morgan Department of Radiology and Radiological Science, Johns Hopkins University School of Medicine, Baltimore, MD, United States; ^3^Department of Neurosurgery, Brigham and Women's Hospital, Harvard Medical School, Boston, MA, United States; ^4^Department of Neurosurgery, University of Alabama, Birmingham, AL, United States; ^5^Medical Scientist Training Program, University of California, Los Angeles, Los Angeles, CA, United States; ^6^Senior Research Scientist, Uber AI Labs, San Francisco, CA, United States; ^7^Department of Neurosurgery, Goethe University, Frankfurt, Germany; ^8^Department of Surgery, Iranian Hospital, Dubai, United Arab Emirates; ^9^Department of Mechanical Engineering, Arak University of Technology, Arak, Iran; ^10^Division of Pediatric Hematology and Oncology, Department of Pediatrics, University of Alabama, Birmingham, AL, United States

**Keywords:** glioma, artificial intelligence, neural network, deep neural network, convolution neural network, support vector machines

## Abstract

**Purpose:** Artificial intelligence (AI) has accelerated novel discoveries across multiple disciplines including medicine. Clinical medicine suffers from a lack of AI-based applications, potentially due to lack of awareness of AI methodology. Future collaboration between computer scientists and clinicians is critical to maximize the benefits of transformative technology in this field for patients. To illustrate, we describe AI-based advances in the diagnosis and management of gliomas, the most common primary central nervous system (CNS) malignancy.

**Methods:** Presented is a succinct description of foundational concepts of AI approaches and their relevance to clinical medicine, geared toward clinicians without computer science backgrounds. We also review novel AI approaches in the diagnosis and management of glioma.

**Results:** Novel AI approaches in gliomas have been developed to predict the grading and genomics from imaging, automate the diagnosis from histopathology, and provide insight into prognosis.

**Conclusion:** Novel AI approaches offer acceptable performance in gliomas. Further investigation is necessary to improve the methodology and determine the full clinical utility of these novel approaches.

## Introduction

Gliomas are the most common primary intracranial neoplasm (1), and comprise 1.8% of human malignancies and 2.3% of cancer deaths (2). World Health Organization (WHO) histopathological classification includes four grades: grades I and II are considered low-grade gliomas (LGG) and grades III and IV (glioblastoma-GBM) are considered high-grade gliomas (HGG) (3, 4). The resilience of gliomas derives from their infiltrative behavior, aggressive biology, genomics, and presence of the blood-brain barrier which reduces the effect of systemic chemotherapy (5). Treatment planning entails initial diagnosis, degree of infiltration, localization and segmentation, genomics, cell biology and clinical/imaging data. Post-treatment evaluation focuses on tumor progression/recurrence. Traditionally, these data are compiled manually by skilled physicians. In the future, artificial intelligence (AI) will augment clinical decision making in the management of oncologic patients, and usher in an era of personalized medicine. An example today is IBM Watson for oncology, a prototypic cloud-based AI, which helps physicians with treatment planning by analyzing extensive clinical, genetic and imaging databases (6–8).

Novel AI approaches such as deep learning (DL), neural networks (NN) and convolutional neural networks (CNN), have facilitated automated extraction of salient clinical data for treatment planning and post-treatment monitoring. Herein, we present a succinct description of foundational concepts of AI approaches and their relevance to clinical medicine. This is geared toward clinicians without computer science backgrounds. Also, we review novel AI approaches in the diagnosis and management of glioma. Finally, we discuss the future impact of AI on glioma diagnosis, genomics, perioperative planning, prognosis and post-treatment surveillance and its future challenges.

## Artificial Intelligence

Succinctly, AI aims to create processes that analyze their environment and perform actions to optimize success toward a pre-determined goal. Machine learning is a sub-type of artificial intelligence focused on developing algorithms that can identify patterns within data without explicit specification. These algorithms can be classified into *supervised* and *unsupervised* learning (9). In supervised machine learning, the algorithm is trained on a human-labeled dataset, then the algorithm provides classification or regression on unlabeled data (e.g., for prediction of clinical outcomes). The rate-limiting step is a large human labeled dataset. The most common supervised machine learning techniques are linear and logistic regression, support vector machines (SVMs), naive Bayes, decision trees, and random forest methods. In unsupervised machine learning, algorithms identify hidden patterns for unlabeled datasets that are unknown to humans. The most common unsupervised machine learning methods include K-means, mean shift, affinity propagation, hierarchical clustering, Gaussian mixture modeling, and self-organizing maps. In machine learning, the input data for the above-mentioned algorithms are called features, which can be numerical or nominal values.

**Figure 1:**
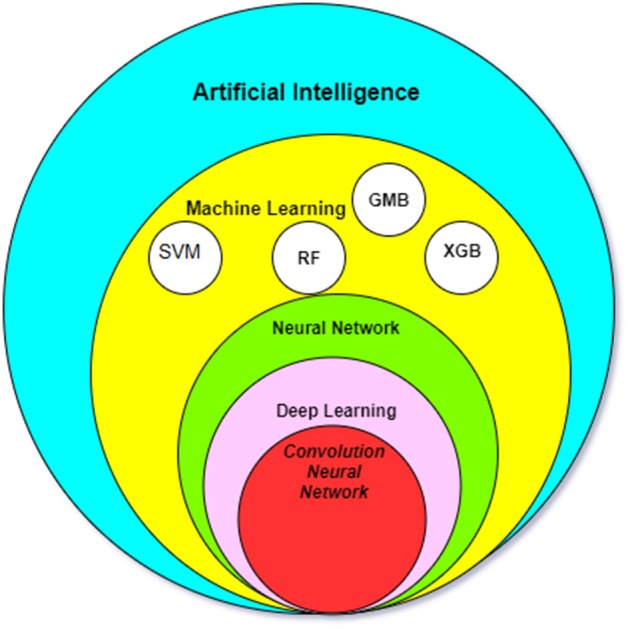
The relationship between the most common AI methods in medicine. SMV, Support Vector Machine; RF, Random forest algorithm; GBM, Gradient Boosting Machines; XGB, XGBoost.

As an example, common features of neuroimaging data are location, size, shape, and signal intensity. In addition to these features, machine-learning algorithms are able to develop new inputs which are not readily visible to human eyes, including texture information, signal intensity gradient and skewness. Two main machine-learning algorithms, the SVM and the artificial neural network (ANN), have been applied to analysis of imaging data (Figure 1).

**Figure 2:**
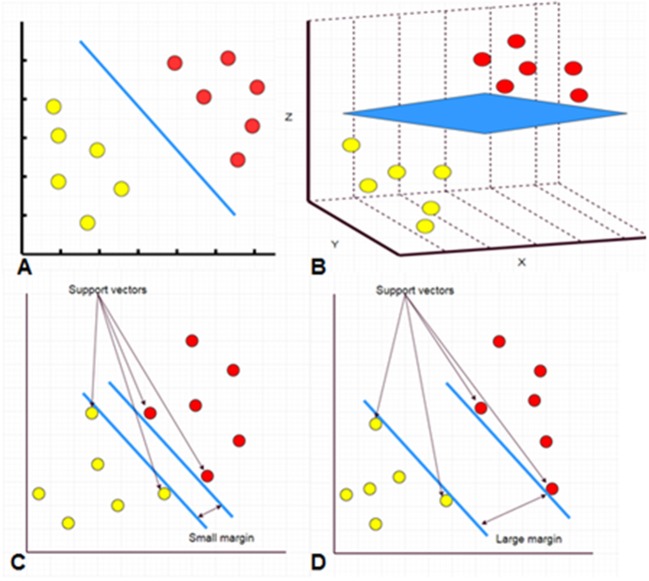
A hyperplane separating two classes of data points in 2D space (**A**, blue line). A hyperplane separating two classes of data points in 3D space (**B**, blue sheet). Separation in more dimensions is also performed but it is difficult to be presented on a 2D manuscript. The support vectors are data that are closer to the hyperplane. The larger the margin between the hyperplanes, the better the classification **(C,D)**.

**Figure 3:**
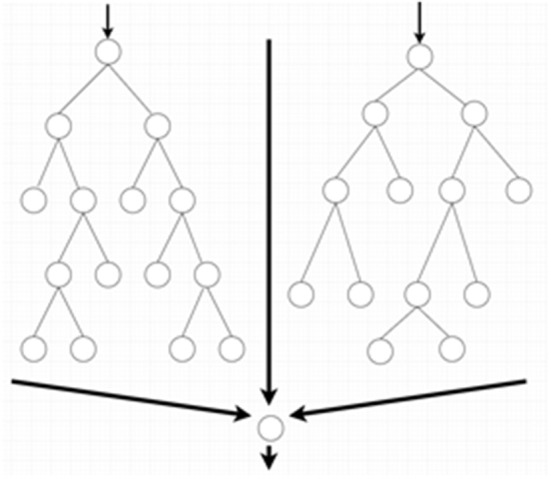
Random forest algorithms by developing multiple decision trees and merging them get more accurate predictions.

SVM and random forest are relatively simple supervised machine learning algorithms that are useful for classifying an object into different categories. The SVM algorithm designs a decision surface in a high-dimensional space (hyper-plane) (Figure 2). SVM works well when the margin of separation between classes is maximized (10). Random forest algorithms develop multiple decision trees and merge them together to get a more accurate prediction. It is one of the most common AI algorithms and can be used for both classification (dividing pieces of data into different categories) and regression (predicting a quantitative response from a predictor variable) (Figure 3).

**Figure 4:**
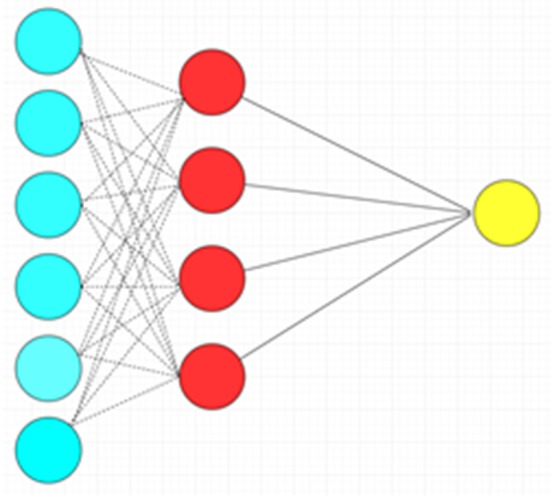
Artificial neural network with a single hidden layer. There is complete connection between layers. Blue circles: Input layer. Red circles: Hidden layer. Yellow circle: Output layer.

The ANN is a more complex machine learning algorithm with many variations that in rough terms attempt to mimic the functionality of biological neural networks. This algorithm is consistent with different nodes of input, hidden and output layers, each layer with a more abstract level of processing compared to the one prior. In the classical variant of ANN known as the multilayer perceptron, the nodes (“neurons”) within each successive layer are all connected to each other between the layers. Each neuron takes the inputs (weighted individually) from the previous layer and performs relatively simple mathematical operations to produce an output. The network can be trained (e.g., to use different weighting schemes) to fit a particular dataset by the use of learning algorithms. The ANN is very flexible for handling different types of data, but it is prone to data-overfitting and requires vast computational resources (Figure 4) (10). Another important disadvantage to neural network approaches is the lack of transparency in the “hidden layers” of neural networks, and the logic behind the mathematical transformations of each layer may not be readily understood. This is in contrast to other AI methods such as random forests, which allow full transparency due to access to the whole “tree” of information (although the decision trees can be rather complex). The lack of transparency inherent to some neural networks has been likened to a “black box”: the clinician cannot fully appreciate how exactly the AI arrives at a solution.

**Figure 5:**
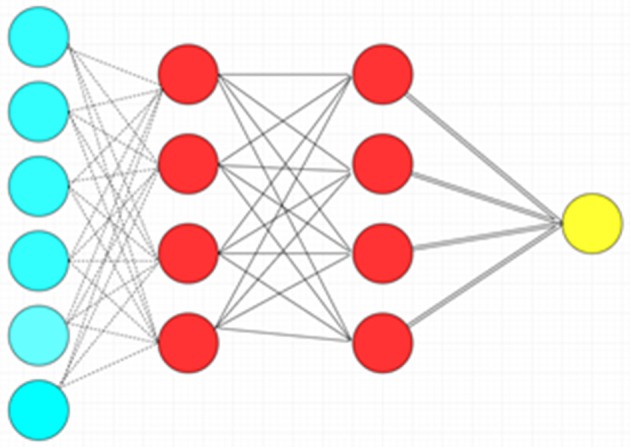
Deep feedforward neural network with two hidden layers. Blue circles: Input layer. Red circles: Hidden layer. Yellow circle: Output layer.

Deep learning is a variant of ANN that adds complexity by using multiple (“deep”) layers of an artificial neural network. The capability to implement deep learning methods using graphics processing units (GPU) and theoretical work over the past decade revived interest in neural networks and demonstrated the sophisticated capabilities of machine learning across many applications. The architecture of a neural network can vary in its complexity, resulting in a wide assortment of ANNs that can be used in deep learning. The standard neural network is termed “feedforward” as the hierarchy of data flows in only the forward direction (Figure 5).

**Figure 6:**
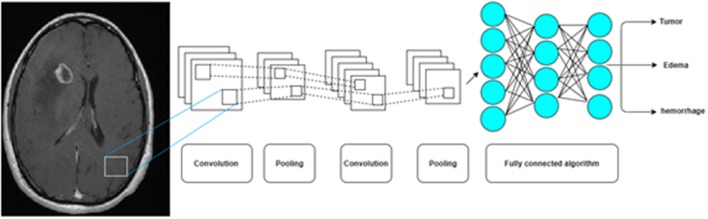
A Convolution Neural Network (CNN) by multiple pooling and convolution steps before a deep neural network is now the most common AI algorithm for image analysis.

The convolution neural network (CNN) is another type of ANN in which imaging data is processed into reduced representations of features using mathematical transformations (most fundamentally, kernel convolution, followed by additional methods for dimensionality reduction), thereby enabling efficient deep learning. These and other innovations have improved the abilities of machine learning in the field of image processing (Figure 6) (10).

## Transfer Learning

Transfer learning is a machine learning method in which an already developed model for a task is reused as the starting point for a model on a second task. The advantage of using these pre-trained models is reducing the time of training. Currently, many of these pre-trained models are provided by major AI companies (e.g., GoogLeNet and AlexNet) which can be used to develop new AI applications.

## Performance Analysis

As with other medical tests, AI algorithms can be evaluated by common biostatistical parameters, including sensitivity, specificity, positive/negative predictive values, and accuracy. Besides these parameters, performance can be evaluated with more dedicated tests. One of the most commonly used statistical tests for analyzing the performance of “classification modules” is the Receiver Operating Characteristic (ROC) Curve which shows the performance of a classification model at all classification thresholds. This model mainly depends on the true positive and false positive rates. In this test, the Area Under the ROC Curve (AUC) is the main indicator of the classification algorithm, represented as a value between 0 and 1. The closer this number to one, the better the performance of the classification algorithm. The “Dice score” is another common test used to evaluate the performance of image segmentation algorithms. This test compares a human-segmented image with the AI-segmented one and measures how similar they are. It is the size of the overlap of the two segmentations divided by the total size of the two objects. More sophisticated algorithms will require increasingly intricate metrics for evaluation of their performance and assessment of the suitability for clinical application.

## Application of AI in the Management of Gliomas

### Grading Prediction by Imaging

Predicting tumor grade on imaging using AI is approaching and may play an important role in future practice. The differentiation of LGG and HGG is crucial for treatment planning and prognosis. Traditionally, this task has been accomplished using anatomic images. LGGs (WHO grade I & II) usually do not show contrast enhancement and are without increased perfusion on MR perfusion sequences. Grade III gliomas may show punctate nodular enhancement and mildly increased perfusion. GBM (WHO grade IV) shows avid rim enhancement, marked hyper-perfusion and central necrosis (11–13). While these coarse imaging features that are appreciated by clinicians have reasonable predictive value, AI algorithms that can assess features not readily observed by humans may improve the differentiation of LGG vs. HGG.

Different techniques of machine learning applied to various imaging modalities have been studied for glioma grading. On the simple end of the spectrum, logistic regression classifiers have been developed to predict grade based on texture features in 34 GBMs and 73 LGG. This model achieved accuracy of 93%, a sensitivity of 97%, a negative predictive value of 99%, and an AUC of 0.94 in differentiating LGG vs. GBM (12). In comparison to LGG, HGGs have more complex anatomical morphology and BOLD-fMRI features. SVM has been employed to predict glioma grading based on resting-state functional MRI images, although these are not typically acquired in clinical practice. This model achieved accuracy of 89% in grading prediction (14). In another study, the SVM classifiers were developed to diagnose low-grade vs. high-grade and grade III vs. IV gliomas. SVM models detected 30 and 28 optimal features for classifying LGGs from HGGs and grades III from IV, respectively. The AUC was 0.987 for classifying LGGs from HGGs, and 0.992 for classifying grades III from IV (15). In a study of 130 gliomas, an artificial neural network used 41 features based on 2D T1-weighted MRI images. The algorithm was able to differentiate LGG vs. HGG with an accuracy of 90.3%, mean sensitivity of 87.8% and mean specificity of 92.5%. The AUC was 0.9486 (16). Ranjith et al. (17) differentiated LGG (only WHO grade II) from high-HGG (WHO grade III and IV) using MRI spectroscopy and machine algorithms in 38 patients (including multilayer perceptron, support vector machine, random forest, and locally weighted learning). They reported a AUC of more than 0.8 in three algorithms; with the best AUC using the random forest algorithm (0.911) and the best sensitivity using locally weighted learning (86%).

By using deep learning algorithms and transfer learning, researchers were able to predict glioma grading from T1-weighted, contrast-enhanced images before surgery in 113 patients with the high performance of the GoogLeNet and AlexNet software. They showed that the performance of transferred learning algorithms outperformed the trained CNN algorithm. Overall, the performance of GoogLeNet was better than AlexNet. The mean value of validation accuracy, test accuracy and test AUC of GoogLeNet was 0.867, 0.909, and 0.939, respectively. For AlexNet, the mean value of validation accuracy, test accuracy and test AUC were 0.866, 0.855, and 0.895, respectively (18). In this study, grades II and III were considered as low-grade and grade IV as high-grade.

In summary, these studies suggest grade prediction based on imaging features is feasible by AI algorithms. In comparison to humans, the machine learning algorithms are capable of using large numbers of imaging features which may improve the grading prediction power. It is still unclear if automated grading can change clinical management. Comparison of the various studies is difficult since a universal definition of HGG and LGG by imaging characteristics was not used. In addition, it is unclear which machine learning algorithm works best. In one study, 25 common machine-learning algorithms were compared to predict glioma grading; the SVM exhibited superior performance to other classifiers. In this study, grade II, III and IV were considered as high-grade which makes comparison with other studies challenging (19).

### Genetic Information

Clinicians have attempted to estimate histopathologic features and glioma genomics, which provide important classifications of the tumor that guide treatment and prognosis, from non-invasive imaging studies with the use of AI. Traditionally, the diagnosis of glioma depends on the histopathological examination, but this practice has changed in recent decades due to advancement in genetic studies. Current WHO classification of CNS malignancies is in part based on genetic mutations. For example, the classification of astrocytomas and oligodendrogliomas depends on the mutation status of *IDH1*/2, *ATRX* loss, and *p53* mutations (in astrocytomas), and co-deletion of 1p and 19q (in oligodendrogliomas). In other tumors, such as midline gliomas, the presence of the H3K27M mutation can redefine the grade of a tumor from a low-grade (by histo-anatomic diagnosis) to a grade IV entity known as “diffuse midline glioma, H3K27M-mutant.” Through the advancement of imaging modalities, it is possible to predict the pattern of genetic mutation based on the evaluation of the radiologic features. AI has a major impact on this “radiomics” approach. Radiomics describes a broad set of computational methods that extract quantitative features from radiographic images that are often beyond the ability of human eyes to see (20, 21). Notably, most of the radiomics approaches depend on the hand-engineered features including size, shape, location, texture, intensity and peri-tumoral features; however, these approaches are limited in the information they can evaluate. In this context, several researchers have developed machine learning-based algorithms to predict genetic mutations from the imaging. So far, most of the research was dedicated to predict the isocitrate dehydrogenase (IDH) mutations, O (6)-methylguanine-DNA methyltransferase (MGMT) promoter methylation, and co-deletion of chromosome arms 1p/19q with better results in comparison to hand-engineered radiomics (22). These genetic features are often associated with better treatment response and survival rates (23–31).

Akkus et al. (32) trained a CNN algorithm based on T1 post contrast and T2 sequences and was able to predict the co-deletion of chromosome arms 1p/19q with a sensitivity of 93.3%, specificity of 82.2%, and accuracy of 87.7%. By using a residual convolutional neural network for each MR sequence including the FLAIR, T2, T1 pre-, and post-contrast, other researchers predicted the presence of IDH mutation with an accuracy of 85.7% without including the patients' age and 89.1% after including the age factor (33). Wu et al. (34) utilized 126 glioma patients, 704 radiomic features and eight classical machine learning methods in an effort to predict IDH genotype in diffuse gliomas before surgery. They reported high predictive performance with a random forest algorithm (accuracy 0.885 ± 0.041, AUC 0.931 ± 0.036) but low predictive performance with a neural network (accuracy 0.829 ± 0.064, AUC 0.878 ± 0.052). They concluded that a random forest algorithm is suitable for IDH genomic prediction before surgery of glioma. Random forest algorithms also have been tested on grade III and IV gliomas for prediction of IDH mutation with an accuracy of 89% and AUC of 0.9231 (35). Deep learning based radiomics algorithms has been used to extract data from imaging for prediction of IDH1 mutations in LGG with 6 convolutional layers of 4,096 neurons resulting in an AUC of 92% (36). Other researchers have developed a machine learning based algorithm to estimate the chance of IDH1 mutations in LGG with an AUC of 0.95 (34).

By developing deep learning models, researchers were able to predict the chance of MGMT methylation in GBM with accuracy as high as 95% (37, 38). In a study on archive images of patients with LGG and HGG, researchers developed a deep learning convolutional neural network and were able to estimate codeletion of chromosome arms 1p/19q, IDH1 mutation, and MGMT methylation with an accuracy of 92, 94, and 83%, respectively (39). Liu et al. (40) have used the combined model of CNN features and a support-vector-machine classifier to automatically predict genotypes of midline gliomas (H3 K27M mutation) with an accuracy of 94.8% to predict the mutation.

In summary, prediction of tumor genomics from imaging data is feasible by application of AI algorithm radiomics. The advantages of AI are its ability to detect multiple radiologic features which are too numerous and too subtle for the human eye. Most reported algorithms achieved high performance with accuracy above 80–90%. So far, the majority of genetic studies utilize imaging data to predict gene mutations. Machine learning algorithms have also been used to extract data from genetic databases. These algorithms were able to classify the patients with GBM into different clusters and predict the prognosis and treatment response (41). Accuracy of about 89% has been reported with the application of neural network-based classifiers to help differentiate the transcriptional subtypes of GBM (e.g., mesenchymal, classical, proneural, and neural subtypes) (42). AI algorithms are a promising approach to help analyze gene expression and predict the histopathology of GBM subtypes.

### Pre-operative Planning

The 3D volumetric measurement of the viable/enhancing tumoral component and peripheral edema is essential for surgical planning and post-operative follow-up. Manual 3D segmentation methods are time-consuming (43). AI has been used for tumor segmentation (44, 45). To differentiate voxels representing viable neoplasm vs. edema vs. normal brain tissue, several machine-learning algorithms have been used. So far, the most promising techniques are SVM, random forest and CNNs. The CNN models have the best performance (22, 46–49). In one study a tumor localization network (a fully convolutional network in conjunction with transfer learning technology) was used to localize the tumor (50). This two-step protocol was faster than and at least as accurate as the prior reported methods in the literature (50). A 3D U-Net CNN has also been used for automated segmentation of gliomas on 18F-fluoroethyl-tyrosine (18F-FET) PET with 88% sensitivity, 99% specificity, a 78% positive predictive value, and a 99% negative predictive value (51). In another study, a multipathway convolutional neural network and fully connected conditional random field were implemented for 3D FLAIR images for segmentation of a LGG with a Dice similarity coefficient of 0.85 (52). Additionally, SMV has been used to differentiate viable HGG from peri-tumoral edema in a study containing 9 patients with HGG: the SMV was able to differentiate viable “non-enhancing” tumor from peripheral edema with a misclassification error of 8.4%. When SVM output was smoothed using a mean filter, the misclassification error was reduced to 2.4 % (53). Automated algorithms have been developed by using random forest models combined with voxel texture features on contrast-enhanced T1 and FLAIR MRI for glioma segmentation. This model had an overall moderate accuracy with better performance to segment high-grade enhancing neoplasm and edema in comparison to non-enhancing LGG or necrosis (54).

Gliomas, specifically GBM, are infiltrative neoplasms. They often invade the tissues beyond the enhancing area on MRI. Given the fact that surgical resection of glioma includes resection of mostly enhancing tissue, non-enhancing tumor can be left behind. AI can help physicians predict the location of subsequent recurrence. Currently, an AI algorithm has been developed to perform this task with AUC of 84% (55).

In summary, different AI techniques can segment tumor, edema and normal tissue. In this context, the CNNs, SVM, and random forest algorithms are promising with at least moderate accuracy.

### Intra-Operative Treatment Planning

Gliomas display an infiltrative behavior. Differentiation of tumor vs. normal tissue is challenging, not only by imaging, but also during surgery. In this context, intraoperative MRI scanners have been used at some oncologic centers for this purpose, although their application is limited by their cost, availability and logistics. An interesting application of AI has been introduced to help neurosurgeons resect the maximum amount of tumor and minimum amount of normal tissue. In this technique, deep learning methods are used to analyze the images from hyperspectral imaging (a non-contact, non-ionizing, label-free and intraoperative imaging modality) during surgery with accuracy about 80% to differentiate neoplastic tissue from adjacent non-tumoral brain tissues (56).

### Histopathologic Diagnosis

In classical medical practice, pathologists and clinicians usually analyze the histopathologic features of the disease. In some settings, such as during surgery, the amount of time from preparation of tissue to diagnosis may be a limiting factor. The diagnosis depends on pathologist's expertise and is critical for the management of the patient, both intraoperatively and for subsequent treatment (57–59). Considering these aforementioned restrictions, computational histopathology is becoming more popular (60, 61). The application of AI to pathology has been aided by the advent of slide scanners, which can convert microscopic slides to high-quality image files. Once digitized, the slides are amenable to computation. In the future, AI methods may assist neuropathologists with identifying characteristics of a tumor and allow for interpretation of subtle histological features that cannot be easily appreciated by humans.

Microscopically, glioma biopsy specimens have the appearance of a collection of variably pleomorphic neoplastic glial cells, sometimes mixed with normal brain tissue. Different tissue architecture (e.g., mixed mesenchymal and glial areas in gliosarcoma) may be present. Immune cells, blood vessels, and areas of necrosis may also be seen. The morphology of the neoplastic glial cells can assist in the classification of the tumor; in addition, the observation and frequency of mitotic figures are important for determination of HGG vs. LGG to patients. Additional pathological features, such as the presence of vascular endothelial hyperplasia or palisading necrosis, also play a role in defining HGG. The complex picture provided by sometimes limited specimens provides a rich substrate for AI approaches for analysis.

While usually readily identifiable, non-neoplastic cells may sometimes mimic glioma cells, confounding their differentiation. For example, reactive astrocytes may appear similar to neoplastic astrocytes, but are differentiated by the cellular spacing and nuclear atypia; likewise, prior radiation treatment may result in abnormal or bizarre-appearing cells that can be challenging to distinguish from GBM recurrence. Abas et al. (62) used multiple machine learning methods, such as the SVM and decision trees, to segment and classify these cells on smear preparations of glioma. SVM and random forest algorithms have also been used for nucleus segmentation on histopathologic images from a glioma database and were more than 98% accurate for classification of LGG or HGG (63). Beyond cytology, Yonekura et al. (64) reported that the CNNs could extract significant features from GBM histopathology slide images with an accuracy of about 98%. SVM algorithms have been used for diagnosis and glioma grading (grade II, III, and IV) on slide images with promising accuracy of about 90% (65). In a recent study, a machine learning technique (Google Inception V3 convolutional neural network) was used for diagnosis of glioma. The haemotoxylin and eosin (H&E) stain images from 50 normal brain, 45 LGG, and 59 HGG were analyzed. The accuracy of this algorithm was 100% for the diagnosis of HGG vs. normal brain and 98% for diagnosing glioma (LGG or HGG) vs. normal brain (66).

In addition to diagnosis and grading, AI can help to predict prognosis by combining features from paraffin-embedded tissue specimens and molecular pathology, such as mutations in IDH1/2 genes and codeletion of chromosomes 1p and 19q. In a study on 769 gliomas including LGGs (grade II and III) and HGGs (grade IV), the CNN was able to predict survival rate with accuracy equal to the manual histologic-grade baseline models (67). While the application of AI to the histopathology of gliomas is only in its infancy, such approaches promise to aid in the evaluation of large tissue specimens and improve diagnostic accuracy for patients in the near future. Pathologists may benefit from segmentation algorithms which rapidly highlight and classify cells (such as mitotic figures), or which can define regions of increased cellularity. In the future, it is not inconceivable that AI-assisted methods may define new pathologic subsets of gliomas with their own characteristic responsiveness to treatments and prognosis, and therefore open new avenues in the pathologic examination of gliomas.

### Radiation

AI has the potential to positively impact the field of radiotherapy. Patient selection, simulation, treatment planning, quality assurance, and follow-up are the most important steps in radiotherapy. Simulation of glioma is less challenging than other visceral malignancies with respiratory motions. Treatment planning includes identification of glioma and adjacent organs which may be damaged by radiation, Organs At Risk (OAR). Segmentation of tumor and OARs are traditionally performed by automated software with atlas-based segmentation tools techniques. The atlas-based segmentation tools are accurate for segmentation of high contrast organs (e.g., lung and bone) but they are suboptimal for tissues with close densities (soft tissues). In this context, machine and deep learning techniques approaches may improve the segmentation and treatment planning (56).

### Post-treatment Follow-Up

Differentiation of post-treatment changes including radiation necrosis and pseudo-progression vs. true tumor progression/recurrence is a very common challenge in neuroradiology. This task is difficult for anatomic images; therefore, many advanced imaging techniques have been proposed (e.g., MR spectroscopy, MR perfusion and PET with difference tracers) with persistent uncertainty. Very few studies are available involving application of AI to differentiate post-treatment changes vs. CNS tumor progression. The SVM classifier has been trained to diagnose pseudo-progression vs. recurrence in patients with glioma treated with surgery and chemotherapy. In this study of 31 patients, the sensitivity and specificity of the classifier for pseudoprogression was 89.91 and 93.72%, respectively with AUC of 0.94; of note, the best predictor image sequences were DWI and MRP (68). A CNN has been developed to differentiate true vs. pseudo-progression in patients with GBM status post resection and chemo-radiation with acceptable performance and an AUC of 0.83 (69). In one study, AI was applied for this task with a high performance of SVM classifiers outperforming two expert neuroradiologists with an AUC for FLAIR sequence equal to 0.79. The main limitation in this study is the fact that researchers included the primary and metastatic CNS tumors in combination (70).

### Outcome Prediction

There are many models to predict the overall survival rate in patients with glioma. Most of them are based on clinical data, genetic information, and imaging. Recently, many AI algorithms have been developed to predict survival rate in these patients, and potentially have superior accuracy compared to conventional methods (71). Nie et al. (72) have developed a multi-module and multi-channel deep survival prediction model for glioma using CNNs to analyze MRI images (including the T1 post contrast, DTI and resting state functional MRI) in association with a SVM (which contains information on tumor histology, tumor size and patients age) to predict the overall survival of patients with glioma with an accuracy of 90.66%. In another study, the Pathway-Associated Sparse Deep Neural Network (PASNet) outperformed other models to predict the outcome of GBM with an AUC of 0.66 (73). Machine learning by means of SVM in combination with whole-tumor rCBV histogram analysis has been used for outcome prediction of glioma with an AUC of 0.7 to 0.8 (74). Additionally, SVM algorithms predicted the outcome of gliomas from MR images with an 80% accuracy of 80% (61). SVM has been used to analyze the anatomic (DTI) and functional (rs-fMRI) data to predict good outcomes (>650-day survival) vs. poor outcomes (<650-day) in high-grade glioma with an accuracy of 75% (75). By developing a convolutional neural base network, Lao et al. (76) predicted the survival rate of GBM patients with a C-index of 0.71, which was subsequently improved after inclusion of the clinical data. C-index is equal to the AUC and ranges from 0.5 to 1. Values above 0.7 indicate a good test.

In summary, the overall accuracy of machine learning methods to predict glioma outcomes from imaging and clinical data approaches 80%, as evident on the meta-analysis done by Sarkiss and Germano (71) on 29 studies (including glioma and brain metastasis). In the future, AI will help to integrate data from disparate fields (clinical examination, imaging, and pathology) to guide treatment and prognosis. One major uncertainty about these prognostic algorithms is the heterogeneity of the patient population. One recent study of machine learning on a small heterogeneous population of glioma using 21 features (demographic, clinical, genetic, histopathologic data) achieved accuracy of <70% (77).

## Future Challenges

AI may demonstrate a crucial role in patient selection for clinical trials, specifically with biomarkers and radiomics. Classically, in glioma clinical trials, the most common biomarkers are the status of MGMT promoter methylation and IDH mutation but on the horizon are radiomic markers which can predict the treatment response to a particular treatment. This would help improve clinical trial designs with more finely targeted inclusion and exclusion criteria and potentially improve trial outcome (6, 78–80). Additionally, there are commercially available AI techniques for extraction of patient data, to link them to large databases and to select the best trial and medication for those specific patients (81).

Several challenges must be addressed before the adaptation of AI in oncology and specifically, the management of glioma. Developing accurate AI needs large standardized and annotated data sets and high-quality ground truth data. The multi-institutional nature of most clinical trials for gliomas complicates the ability to acquire uniform data sets (82). Although AI algorithms use numerous features for decision making, their analysis processes are not always readily understood to humans, so many of the previously described AI algorithms are not amenable to be re-created by other investigators. There is ongoing research to solve this “black-box” nature of AI, and in the future, may help to follow otherwise impenetrable processes step-by-step in transparent algorithms (39).

Generalizability of the AI algorithms is one of the major challenges preventing their widespread clinical adaptation. So far most of the AI applications in oncology and gliomas have been trained on relatively small patient populations. Performance of an AI algorithm developed on a small population is not optimal, especially for the large and heterogeneous population of gliomas (83). In addition, the inclusion criteria in the aforementioned glioma studies were very heterogeneous. Before the clinical adaptation of an algorithm, it is critical to have a universal definition for LGG and HGG. Large, standardized datasets from multiple institutions with clinical, neuroimaging, and neuropathologic data that cover diverse patient populations are needed to fully realize the power of AI for the diagnosis and treatment of gliomas.

Given the inchoate stage of development for AI in gliomas, there are no comprehensive cost-benefit studies or prospective studies to confirm that AI can improve patient outcome. Another obstacle against universal adaptation of AI in oncology and gliomas is clinician-engineer interaction. Currently, physicians receive very little training in computer/data science and most of the computer scientists are not familiar with the complexity of clinical patient management (81).

Thus, the abovementioned challenges, as well as many unanswered legal and ethical questions, must be addressed before the adaptation of AI in the daily practice of oncologic centers (84).

## Conclusion

Many AI approaches have been created to help with glioma management. AI techniques have been developed to predict grading from imaging data; survival rates from clinical, genetic and imaging data; and molecular genetics from imaging data. AI techniques have also been developed to automate diagnosis from histopathologic slides to segment tissues for surgical planning and to monitor the patients after treatment. Most of these techniques suggest acceptable performance, but the application of AI to glioma diagnosis and treatment has only now begun in earnest. The promise and performance of these techniques in daily clinical practice and their effect on patient outcomes warrant further development.

## Author Contributions

HS, OS, ES, and MB contributed conception, design of the study, and wrote the first draft of the manuscript. HS organized the database. JB, GE, JC, PS, GC, FG, AS, and GF wrote sections of the manuscript. All authors contributed to manuscript revision, read, and approved the submitted version.

### Conflict of Interest Statement

JB has positions/equity in CITC Ltd and Avidea Technologies and is a member of the Scientific Board of Advisors for POCKiT Diagnostics. PS was employed by Uber AI Lab. The remaining authors declare that the research was conducted in the absence of any commercial or financial relationships that could be construed as a potential conflict of interest.
